# 508 Retrospective Review of Early Pulmonary Mechanics After Inhalation Injury

**DOI:** 10.1093/jbcr/irad045.105

**Published:** 2023-05-15

**Authors:** Eric Lam, Rita Frerk, Theresa Chin, Kimberly Burton, Victor Joe, James Jeng

**Affiliations:** UCI Medical Center, Orange, California; UCI Medical Center, Orange, California; UCI Medical Center, Orange, California; UCI Medical Center, Orange, California; UCI Medical Center, Orange, California; UCI Medical Center, Orange, California; UCI Medical Center, Orange, California; UCI Medical Center, Orange, California; UCI Medical Center, Orange, California; UCI Medical Center, Orange, California; UCI Medical Center, Orange, California; UCI Medical Center, Orange, California; UCI Medical Center, Orange, California; UCI Medical Center, Orange, California; UCI Medical Center, Orange, California; UCI Medical Center, Orange, California; UCI Medical Center, Orange, California; UCI Medical Center, Orange, California; UCI Medical Center, Orange, California; UCI Medical Center, Orange, California; UCI Medical Center, Orange, California; UCI Medical Center, Orange, California; UCI Medical Center, Orange, California; UCI Medical Center, Orange, California; UCI Medical Center, Orange, California; UCI Medical Center, Orange, California; UCI Medical Center, Orange, California; UCI Medical Center, Orange, California; UCI Medical Center, Orange, California; UCI Medical Center, Orange, California

## Abstract

**Introduction:**

Management of inhalation injury is largely supportive and consists primarily of mechanical ventilation, bronchodilators, muscarinic receptor antagonists, and inhaled mucolytics and anticoagulants. The purpose of this study is to examine the early changes in pulmonary mechanics after inhalation injury and see if the use of NT affects lung function.

**Methods:**

Retrospective study was done on Inhalation injury patients over a 12 years period from 01/2009 to 01/2021. Patients’ data collected from their medical records, included patient demographics and outcomes, percentage Total Body Surface Area (TBSA), Carboxyhemoglobin level upon admission, Respiratory Treatments (bronchodilator, Mucolytic and Heparin), Tobramycin treatment, Oxygen requirement, Lung compliance (peak airway pressure & Mean airway Pressure), Mode of Ventilation, total Ventilator Day, and number of Hospital Days. Patients with inhalation injury from 2009-2019 were retrospectively reviewed for treatment of inhalation injury. Univariate analysis and multiple logistic regression were performed using Excel and Stata.

**Results:**

Of 90 patients with inhalation injury, median age was 53.5 (IQR:31-65) with 30% (n=28) women and median TBSA of 15%(IQR:2-38%). The median length of stay was 21 days (IQR:6-47). Median ventilator days was (8.5, IQR:3-21) and median ICU days was 12 (IQR:3-28). After adjusting for grade of inhalation injury, patients who did not receive NT were 2.7 times as likely to get pneumonia compared to patients who received NT (p=0.037). Initial median carboxyhemoglobin was 3.9 (IQR: 2-9.1). Overall mortality was 21%, but the incidence of death increased with grade of injury

The Median Mean Airway Pressures were Day 1: 9.5mmHg, Day 2: 11mmHg, Day 3: 13mmHg, Day 4: 12mmHg

The median peak pressures were Day 1; 20mmHg, Day 2: 21mmHg; Day 3: 22.5mmHg; Day 4: 21mmHg

**Conclusions:**

Patients with inhalation injury are susceptible to Acute Respiratory Distress Syndrome, indicated by a decrease in lung compliance and increase in the patient’s oxygenation support. The lung compliance of these patients being to change resulting in an increase in their Peak Airway Pressure and Mean Airway Pressure within the first 72 hours. Moderate hypoxemia is present in these patients with the progress of the smoke inhalation injury. The usage of NT did not have a direct benefit to the pulmonary mechanics for inhalation injury when used as a prophylactic strategy. Additional research and clinical trials are needed to better understand the effect of inhalation injury on pulmonary mechanics.

**Applicability of Research to Practice:**

Analysis of pulmonary mechanics in patients with inhalation injury can allow clinicians to better tailor ventilator settings to improve clinical outcomes.

